# Shape Fidelity of 3D-Bioprinted Biodegradable Patches

**DOI:** 10.3390/mi12020195

**Published:** 2021-02-13

**Authors:** Mikail Temirel, Christopher Hawxhurst, Savas Tasoglu

**Affiliations:** 1 Department of Biomedical Engineering, University of Connecticut, Storrs, CT 06269, USA; mikail.temirel@uconn.edu; 2 Department of Chemical and Biomolecular Engineering, University of Connecticut, Storrs, CT 06269, USA; Christopher.Hawxhurst@uconn.edu; 3 Department of Mechanical Engineering, Koç University, Sariyer, 34450 Istanbul, Turkey; 4 Koç University Arçelik Research Center for Creative Industries (KUAR), Koç University, Sariyer, 34450 Istanbul, Turkey; 5 Boğaziçi Institute of Biomedical Engineering, Boğaziçi University, Çengelköy, 34684 Istanbul, Turkey; 6 Center for Life Sciences and Technologies, Bogazici University, Bebek, 34470 Istanbul, Turkey; 7 Koc University Research Center for Translational Medicine, Koç University, Sariyer, 34450 Istanbul, Turkey

**Keywords:** biopriting, bioprinter, extrusion, fidelity, cellulose nanocrystal, cellulose nanofiber, alginate

## Abstract

There is high demand in the medical field for rapid fabrication of biodegradable patches at low cost and high throughput for various instant applications, such as wound healing. Bioprinting is a promising technology, which makes it possible to fabricate custom biodegradable patches. However, several challenges with the physical and chemical fidelity of bioprinted patches must be solved to increase the performance of patches. Here, we presented two hybrid hydrogels made of alginate-cellulose nanocrystal (CNC) (2% *w*/*v* alginate and 4% *w*/*v* CNC) and alginate-TEMPO oxidized cellulose nanofibril (T-CNF) (4% *w*/*v* alginate and 1% *w*/*v* T-CNC) via ionic crosslinking using calcium chloride (2% *w*/*v*). These hydrogels were rheologically characterized, and printing parameters were tuned for improved shape fidelity for use with an extrusion printing head. Young’s modulus of 3D printed patches was found to be 0.2–0.45 MPa, which was between the physiological ranges of human skin. Mechanical fidelity of patches was assessed through cycling loading experiments that emulate human tissue motion. 3D bioprinted patches were exposed to a solution mimicking the body fluid to characterize the biodegradability of patches at body temperature. The biodegradation of alginate-CNC and alginate-CNF was around 90% and 50% at the end of the 30-day in vitro degradation trial, which might be sufficient time for wound healing. Finally, the biocompatibility of the hydrogels was tested by cell viability analysis using NIH/3T3 mouse fibroblast cells. This study may pave the way toward improving the performance of patches and developing new patch material with high physical and chemical fidelity for instant application.

## 1. Introduction

Millions of people suffer from tissue loss or organ defects, contributing to over $400 billion per year total healthcare expenses in the United States [1]. Tissue engineering has the potential to help overcome these challenges through the development of regenerative tissues [2,3,4], autologous cells, biodegradable scaffolds, various implants, such as arterial reconstruction [5] and bone regeneration [6], and organ-on-a-chips [7,8,9,10]. Multi-material and multi-functional biodegradable patch research is a promising field. It offers increased functionality, cost-efficiency, and production feasibility with organic and inorganic materials; moreover, they are biodegradable, meaning that they are gradually extinguished from the body after fulfilling their functions. Biodegradable patches have been used for a variety of reasons, including restoring function, facilitation healing, and replacing organs, such as skin or tissues after injury or disease. Biodegradable patches have been used for different applications, including wound healing [11], cardiac reconstruction [12], health monitoring [13], and drug delivery [14]. Their use in wound healing is particularly promising. The skin is the largest organ in the body and is an open door for potentially harmful impairments or injuries, such as acute trauma, burns, surgical defects, or long-term diseases, such as eczema and diabetes [15]. A skin injury larger than 4 cm will not heal without external support [16], often requiring donor skin; however, the availability of donor skin is limited. Biodegradable patches offer a robust alternative solution via the composition of non-toxic and non-allergic materials.

The first biodegradable patches were single- or multi-layered patches with no spatial heterogeneity in planar material properties or functionality [17]. Recently, with the growth of wearable technologies and their emerging applications, electronics have been integrated into patches, and spatially heterogeneous material deposition has been achieved [13,14,15]. From this perspective, 3D (bio)printing can be used for the high throughput fabrication of these patches via multi-material deposition without requiring manual handling or steps. Bioprinting can readily produce custom biodegradable patches that are compatible with specific native tissues, such as external blood vessels and bone, and have the potential to engineer fully functional organs as well as patient-specific tissues. Furthermore, modern applications of patches, such as health monitoring, require complex electronic integration with electronic sensors [13], which can be achieved by 3D (bio)printing at high throughput, potentially in a single step. Thus, 3D (bio)printing technology is expected to be increasingly used to generate patches due to its ability to deposit multiple materials in a single step. Here, we leveraged these unique advantages of bioprinting technology to fabricate free-template, multilayer, and heterogeneous complex patches.

3D bioprinting technologies offer rapid and early wound treatment to avoid aggravation, tissue damages, and hypertrophic scarring for multiple types of wounds, including burn, diabetic, surgical wounds. The 3D bioprinting technologies had been started to use for wound healing and skin regeneration in 2012 using natural bioink collagen, and it reached around 70 published studies by 2020 [18]. Extrusion-based bioprinting is the widely used method [19], and various crosslinking methods have been used, such as chemical crosslinking by calcium chloride [20] and UV light [21,22]. Collagen [23], gelatin [20], and alginate [24] are bioink materials used for wound healing due to their properties, such as similarity to the extracellular matrix (ECM), printability, and biocompatibility. Most of the in vitro studies employed the fibroblast skin cells, such as human dermal fibroblast cells [25] and 3T3 mouse fibroblast cells [21]. In addition to the in vitro study, there are many animal studies conducted for wound healing by using mice [19,25], rats [26,27], and porcine [19].

Fidelity is one of the main aspects of patches influencing their functionality [28,29,30,31,32]. A patch must maintain its shape and structure for some time on the target tissue or organ, then biodegrade after fulfilling its role. Mechanical loading is an important design consideration; for example, patches have been proposed or developed to serve as pressure sensors on vessels [13] and organs and as repair materials for cardiac reconstruction in patients with complex congenital heart defects [12]. In tissues that undergo periodic dilation and constriction, the shape of the patch and its material properties, biodegradability, and electronic components may be affected. It is critical to ensure that a patch maintains its fidelity under the mechanical loading over the targeted time. Depending on the target organ or vessel, patches are intended to stay in the body for various lengths of time. For instance, a patch for monitoring blood flow may stay on a vessel for twelve weeks [13], while a cardiac patch might remain on an organ for ten weeks [33]. In addition, patches are usually prepared at room temperature (22–26 °C) before being applied to the body (37 °C). This temperature difference can affect the fidelity of a patch.

To achieve high fidelity (performance) bioprinted cell-laden patches, one of the most important factors is the hydrogel material (bioink) [34,35,36,37,38]. The hydrogel needs to have enough viscosity to preserve its shape after deposition and must keep its shape post crosslinking. Bioprinting hydrogel has several requirements, including effective printability, biodegradability in vivo, and strong and elastic physical properties for mechanical loading [39]. Alginate is a widely used hydrogel material in bioprinting due to its low-cost, easy and rapid crosslinking with calcium chloride, innate biocompatibility while maintaining high cell viability. Alginate, however, has several drawbacks to its use in bioprinting, including low mechanical properties, low viscosity, slow degradation, and limited printability [40]. This limits its design and use in some applications that required mechanical loading, such as a cardiac implant. To counter this issue, other materials can be added to increase the viscosity and mechanical strength. For example, the structural fidelity of printing with alginate has been increased by blending the alginate with carbon nanotubes (CNT) [41], gelatin methacryloyl (GelMA), hydroxyapatite [40,42], and cellulose [43,44].

Cellulose nanoparticles are one of the most attractive co-materials for tissue engineering. Cellulose nanoparticles have several advantages, including sustainability, biocompatibility, abundance, water-retention, and high chemo-mechanical properties [45,46,47]. Cellulose nanoparticles can also be found in different forms, including cellulose monocrystalline (CNC), which is used for many applications, including reinforcing alginate-based hydrogels [48]. Another form is cellulose nanofibril (CNF), which is used to improve the rheological and mechanical properties of pure alginate with respect to printability and structural fidelity [49]. Of particular interest is one form of CNF, 2,2,6,6-tetramethylpiperidine-1-oxyl radical (TEMPO)-mediated oxidation cellulose nanofibril (T-CNF), which shows excellent printability, mechanical strength, and viscosity within the ideal range [50].

We characterized the concentration of CNC- and CNF-based hydrogels and printing parameters, such as pneumatic pressure, nozzle speed, and line thickness, to achieve the best shape fidelity with our custom-made bioprinter [51]. We also characterized the rheological properties of optimized hydrogels, such as viscosity and storage-loss modulus, and performed mechanical characterization of 3D printed and cross-linked patch samples through mechanical loading. Finally, we conducted a biodegradability and biocompatibility characterization by exposing the patch in a chemical solution that mimics the body and bioprinting mouse fibroblast cells in both bioinks over time. This study provided a foundation to develop new patch materials with high printability, fidelity, and biocompatibility. The fidelity analysis methods presented could open a way to enhance the performance of biodegradable patches that can be implanted for wound healing. This study provided a foundation and framework to identify how and when to assess new patch materials to achieve high printability, fidelity, and biocompatibility.

## 2. Material and Methods

### 2.1. Materials

Sodium alginate powder and calcium chloride (CaCI_2_) were acquired from Sigma-Aldrich (St. Louis, MO, USA). TEMPO (Anionic type) Cellulose Nanofibrils (T-CNF) Slurry (1% *w*/*v* solid in water, Width: 20–50 nm; Length: 0.5 μm–80 μm Surface Group: Carboxyl Hydrophilic) was obtained from Cellulose lab (Fredericton, New Brunswick, Canada). Cellulose Nanocrystals (CNC) spray-dried powder hydrolyzed from wood was purchased from CelluloForce (Montréal, Quebec, Canada). HyClone Dulbecco’s Modified Eagle’s Medium with high glucose (DMEM, GE Healthcare Bio-Sciences AB, Uppsala, Sweden) was purchased from cytiva (Global Life Sciences Solutions USA LLC, Marlborough, MA, USA); Fetal Bovine Serum (FBS, Premium) was obtained from Atlanta Biologicals (Hall County, GA, USA). Penicillin-Streptomycin (Gibco, for mammalian cell culture) and cell viability kit (Invitrogen) for cell viability were obtained from ThermoFisher Scientific (Bohemia, NY, USA).

### 2.2. Bioink Preparation

Alginate and CNC powder were dissolved in Milli-Q water at concentrations of 2% and 4% (*w*/*v*), respectively, and vortexed for homogeneity (Cole-Parmer, CT, USA) for 2 min at 3400 rpm. The CNC concentration was chosen as it gave the best printability [52]. To prepare the Alginate-T-CNF bioink, alginate powder (4% *w*/*v*) was added into the T-CNF solution (1% *w*/*v* solid in water) and then vortexed at least 2 min at 3400 rpm. Best shape fidelity was achieved using 4% alginate and 1% T-CNF. The bioinks were maintained at 37 °C for several hours for rapid dissolving. Prepared bioinks were then loaded into 5 mL syringe barrels. The ionic cross-linker, CaCI_2_ powder, was dispersed in Milli-Q water at 2% (*w*/*v*) concentration by mixing on a vortex at 3400 rpm for 1 min. We abbreviated Alginate-CNC as 2A4CNC and Alginate-T-CNF as 4A1CNF.

### 2.3. Printability and Characterization of Bioinks

**Figure 1:**
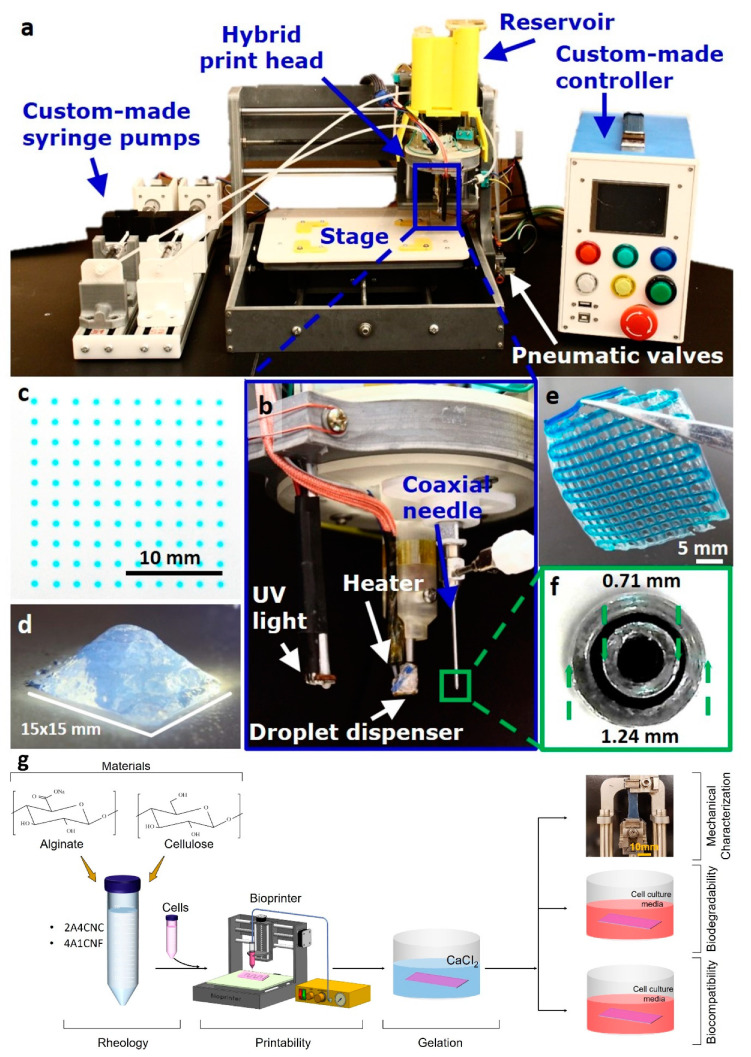
(**a**–**f**) Images of our custom hybrid bioprinter developed previously and given here for completeness. Reproduced with permission from [51]. The custom printer includes a cell-laden droplet dispenser for inkjet 3D bioprinting, a UV light source for photo-crosslinking, and a coaxial head for extrusion-based 3D bioprinting. (**g**) Schematic illustration of the experimental workflow in the current article.

The previously prepared syringe was placed on the printer using a custom-designed 3D printed syringe holder. A 20 × 20 × 1 mm (*l × w × h*) grid pattern was designed on SolidWorks (Dassault Systems SolidWorks Corp., Waltham, MA, USA) and saved as an STL file. The STL file was converted to the G-code as a form of 16 layers’ grid pattern by a slicing engine Slic3r. The nozzle speed was set constant to 7 mm/s for all prints. An air regulator (KLT-982A Auto Glue Dispenser, Tainan City, Taiwan) was used to deposit the bioink on a glass slide with a 30G syringe dispensing tip on the printing platform, as seen in Figure 1b. Different air pressures were tested to determine the best print quality: 8, 12, and 18 PSI for the 2A4CNC bioink and 10, 20, and 30 PSI for the 4A1CNF bioink. All samples were then cross-linked in a 0.18 M calcium chloride solution for two minutes. After crosslinking, samples were imaged by a Carson eFlex digital camera (Carson Optical, Inc., Ronkonkoma, NY, USA). To characterize the line thickness for both bioinks, a one-layer grid pattern was printed and imaged. Line thickness was measured with ImageJ (National Institutes of Health (NIH), MD, US) Optimal air pressures were used to synthesize samples for the rest of the experiment. Different geometries were used for the different experiments: for mechanical characterization and swelling tests, a two-layer geometry (*l × w × h* = 20 × 10 × 0.5 mm) was used; for compression and degradation tests, an eight-layer geometry (*l × w × h* = 10 × 15 × 2 mm) was used. Samples were kept in DI water until needed for characterization experiments.

### 2.4. Rheological Characterization

The rheological properties of bioinks and their components (2% Alg, 4% CNC, 4%Alg, and 1%T-CNF) were analyzed using a rotational AR-G2 rheometer (TA Instrument, New Castle, DE, USA) with a 40 mm in diameter cone palate and a gap of 150 µm. All measurements were done at 25 °C, and samples were allowed to reach equilibrium for one-minute subsequent to adding the sample to the test platform. The shear rate range was varied between 0.01 and 100 s^−1^ to measure the viscosity [49]. Dynamic strain sweep was carried out by using the same cone plate to determine the linear viscoelastic region (LVR). The 1% strain sweep was selected for the test. The frequency sweep was then used to estimate the storage modulus (G’) and loss modulus (G’’) as a function of the angular frequency of 0–100 rad/s [49].

### 2.5. Mechanical Characterization

Samples were mechanically tested using a dynamic mechanical analyzer (DMA Q800, TA Instrument, New Castle, DE, USA) to find the Young’s Modulus in the slope in the initial linear region of the stress-strain curve. Samples (size of *l × w × h* = 20 × 10 × 0.5 mm) were exposed to a ramped force (stress), with a force ramp rate of 0.1 N/min. Simultaneously, resultant deformation (strain) was monitored until the sample failed. Maximum deformation before failure (“Elongation at break”) was also determined. Cyclic loading tests were carried out to assess hysteresis (deformation in response to cycling) over time, mimicking movement in the skin [53]. The cycle tensile was performed in DI water to avoid drying. One hundred cycles were performed; the strain was ramped up and down at 10%/min between 0% and 20%, mimicking the maximum strain on the skin of the human wrist [54]. Hysteresis was calculated from the stress-strain curve of the inside area of loading and unloading [55]. This experiment was carried out at 25 °C.

### 2.6. Swelling Test

3D printed samples were dried at 37 °C overnight in the incubator and then weighed. The dried samples were then submerged in a DI water bath at room temperature for up to 96 h. The swelling was measured at specific times (1, 2, 4, 6, 8, 24, and 96 h), and excess surface water on the sample was removed via blotting before weighing. After measuring was completed, the samples were returned to the DI water bath. The swelling ratio was calculated for samples using the following equation [56]:

(1) $$S=\frac{w_{s}-w_{d}}{w_{d}}\times 100$$

where S is the percentage of swelling of the samples; $w_{d}$ and $w_{s}$ are the dry weight and swollen weight of the sample, respectively. The test was conducted with three different samples for each bioink; error bars reflect standard deviation.

### 2.7. Thermal Analysis

Phase transitions were determined by differential scanning calorimetry (DSC Q20, TA Instrument, New Castle, DE, USA) to estimate the melting temperature. Samples weighing between 4 and 10 mg were placed into hematic aluminum pans, ramped from −50 °C to 250 °C at 10 °C/min, held for 5 min, before being cooled to −50 °C and cycled again under nitrogen atmosphere. The first heating cycle was used for the analysis of the phase transitions of the samples. The heat flow (W/g) was reported as a function of the temperature. The melting temperature was determined as the lower peak point of curves.

Decomposition profiles of 2A4CNC and 4A1CNF bioink samples were determined by thermogravimetric analysis (TGA Q500, TA Instrument, New Castle, DE, USA) to determine the degradation temperature of the hydrogel. Samples were ramped from room temperature to 600 °C at a rate of 10 °C/min under a nitrogen atmosphere. Percentage weight loss was determined as a function of the temperature. Degradation temperature was set as the beginning point on each curve of the TGA line [57].

### 2.8. In Vitro Degradation Test

3D printed samples (*l × w × h* = 10 × 15 × 2 mm) were placed in an environment meant to mimic a living body. Media containing Dulbecco’s modified Eagle’s medium (DMEM) with 10% fetal bovine serum (FBS), kept at 37 °C and 5% CO_2_ in an incubator, was replaced every 2–3 days [58]. Periodically, samples were removed from the incubator, washed with distilled water, freeze-dried for three hours, weighted, and then imaged. Samples were sterilized with UV-light for 20 min before being replaced in the incubator. Normalized weight loss of the samples was calculated with the following equation [58]:

(2) $$Normalized\;Weight\;Loss=\frac{w_{i}-w_{f}}{w_{i}}\times 100$$

where $w_{i}$ is the initial weight; $w_{f}$ is the final weight after being freeze-dried. Degradation tests were conducted three times for each bioink with error bars representing standard deviation.

A compression test was also carried out to investigate the effect of degradation on the material stiffness. Fabricated samples (*l × w × h* = 10 × 10 × 2 mm) were kept in the media in the incubator (at 37 °C and 5% CO_2_) and removed from the media and measured every 5 days over a total span of 30 days. Once removed, samples were washed with distilled water before compression testing at room temperature. Compression tests were performed using dynamic mechanical analysis (DMA). Force was ramped at 1 N/mm to 18 N. Prepared samples were placed on a uniaxial parallel plate with a diameter of 15 mm. Compression was stopped when the sample yielded. Compressive modulus was calculated from the linear region of the stress-strain curve. Three replicates were tested for each time point. Error bars represent standard deviation.

### 2.9. Bioprinting Procedure

Hydrogel biocompatibility was evaluated employing NIH/3T3 mouse fibroblast cells (American Type Culture Collection, Manassas, VA, USA). Fourth stage cells were cultured according to the ATCC protocol over a week to reach the sufficient cell density. Syringes were prepared with 2A4CNC and 4A1CNF hydrogels and sterilized by UV for 20 min. Sterile conditions were maintained, and 3 × 10^6^ cells were then mixed with the hydrogels right before the printing. Nozzle speed was set to 7 mm/s, and air pressures were set at 12 PSI and 20 PSI for 2A4CNC and 4A1CNF, respectively. The bioink mixtures were deposited as a single layer. After printing, the samples were transferred to a well plate, and the CaCI_2_ solution was applied for two minutes to crosslink the bioink. Calcium solution was removed and immediately replaced with warm PBS, followed by two washes with cell media. The bioink samples were kept in cell media at 37 °C for up to 10 days.

### 2.10. Viability Characterization

Cell viability characterization was based on our previous work [51]. Briefly, calcein AM (stains live cells green) and ethidium homodimer-1 (EthD, stains dead cells red) stains were used (Life Technologies, Carlsbad, CA, USA). The stained cells were then imaged with fluorescence microscopy. Samples were washed with PBS, followed by a solution of 1:2000 calcein and 1:500 ethidium homodimer in PBS. Each sample was incubated for 15 min in the staining solution. Images were captured in six different focal planes in the *z*-axis over a range of 100 μm. The six images were combined into a z-stack, and the maximum value of each (x,y) pixel across all six planes was used to create a z-projection image for each channel separately to quantify the cell viability. From each image, the “find maxima” function in ImageJ (National Institutes of Health (NIH), MD, US) [59] was used with a noise tolerance value of 20 to identify local maxima in the image. The local maximum in the green-channel (calcein) was taken as a live cell, and each local maximum in the red-channel (EthD) image was taken as a dead cell. The cell viability of each image was calculated using:

(3) $$Cell\;Viability=\frac{live\;cells}{live\;cells+dead\;cells}$$

The viability was averaged across several images from two different prints. The composite images shown are pseudo-colored to show both calcein and EthD staining in a single image.

### 2.11. Statistical Analysis

In order to evaluate the statistical significance, one-way ANOVA tests for multiple comparisons and *t*-test for two samples comparison were performed. The analysis was conducted with Microsoft Excel 365 (2020). A value of *p* < 0.05 was considered to state statistical significance. All quantitate data were presented as mean ± standard deviations.

## 3. Results and Discussion

### 3.1. Printability and Shape Fidelity

**Figure 2:**
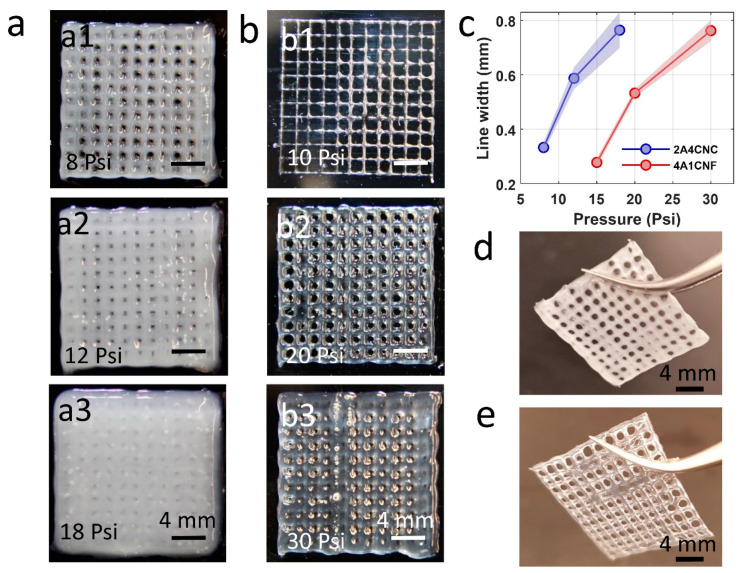
Printability of two hybrid bioink formulations using custom-made bioprinter. (**a**) Representative images of 3D printed 16-layers and 20 × 20 mm square grid pattern for 2A4CNC (2% Alginate and 4% Cellulose Nanocrystals) bioink. Three different air pressures were tested to find optimum pneumatic air pressure. (**b**) Representative images of 3D printed grid pattern with 4A1CNF (4% Alginate and 1% TEMPO oxidized Cellulose Nanofiber) under three different air pressures. (**c**) Line thickness printed by extrusion at various air pressures, showing the effect of air pressure on the filament width. (**d**) Representative images of 2-layers grid pattern after 2 min crosslinking in calcium with an optimum air pressure of 12 PSI for 2A4CNC bioink and (**e**) optimum air pressure of 20 PSI for 4A1CNF.

Assessing the printability of bioinks is important to achieve shape fidelity. Bioink components, concentrations, mechanical properties during 3D printing, such as air pressure, extrusion speed, and nozzle size, are all important factors [60,61,62,63,64,65,66]. In our study, we used a formula of alginate and CNC hybrid bioink as having the best shape fidelity from the literature [52]. We experimentally tested the alginate-CNF bioink formula in terms of printability. We varied the extrusion pressure to find optimum at three different air pressures of 8, 12, and 18 PSI for 2A4CNC and 10, 20, and 30 PSI for 4A1CNF, as seen in Figure 2a,b. Low air pressure does not provide enough force to extrude the hydrogels as the desired pattern, and it causes intermittent printing, as seen in Figure 2a1,b1. High air pressure causes an overflow, which spreads around and fills the gaps in the grind pattern, as seen in Figure 2a3,b3. We determined the optimum air pressure of 12 PSI and 20 PSI for the hydrogel formulations of 2A4CNC and 4A1CNF, respectively. The print accuracy of 2A4CNC hydrogel matches with the previously reported study using the same alginate-CNC hydrogel concentration [52]. The optimized alginate-CNF ratio hydrogel has a better shape fidelity than those reported for either pure alginate or pure CNF [67].

Figure 2c depicts the thickness of a single line as a function of extrusion air pressure, showing the effect of air pressure on filament width. The 2A4CNC line is much thicker than that of 4A1CNF due to the higher viscosity of 4A1CNF. 4A1CNF is more structurally robust and retains its shape after printing compared with 2A4CNC. Figure 2d,e represents the images of the 2-layers grid pattern after 2 min crosslinking in calcium. Those images also demonstrate that the print with 2A4CNC has dull color and print with 4A1CNF has transparent color.

### 3.2. Rheological Properties of Bioink

**Figure 3:**
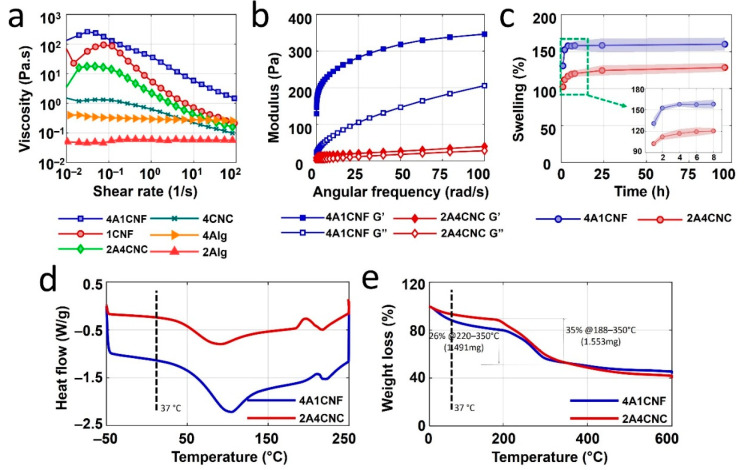
Rheological and thermal characterization of bioinks. (**a**) The viscosity of two hybrid bioinks formulation (4A1CNF, 2A4CNC) and their separate components (4A, 1CNF, 2A, and 4CNC) as a function of shear rate. (**b**) Storage modulus (G’) and loss modulus (G’’) of two bioink formulations (4A1CNF and 2A4CNC). (**c**) Swelling (water absorbance) of dried patches made with these two bioinks over up to 96 h. Differential scanning calorimetry (DSC) analysis results (**d**) and thermogravimetric analysis (TGA) results (**e**) of freeze-dried 4A1CNF and 2A4CNC samples.

The rheological properties of bioinks are characterized to optimize the printability of the proposed bioinks. Figure 3a shows the viscosity measurement results for two hybrid bioinks formulations—4A1CNF, 2A4CNC—and their components—4A, 1CNF, 2A, and 4CNC—as a function of shear rate. Both bioinks have higher viscosities than their components, indicating suitability for bioprinting. The curves for pure 2% and 4% alginate and pure 4% CNC remain relatively linear across shear rates, indicating they are not suitable for bioprinting. The measurements of storage modulus, G’, and loss modulus, G”, of two bioink formulations at frequency sweep of 1% strain are shown in Figure 3b. Storage modulus is higher than loss modulus for both bioink formulations over the angular frequency range of 0–100 rad/s, indicating that both bioinks are solid-like and can hold their shape after printing. These results suggest both bioink can have good shape fidelity. 4A1CNF has a higher storage modulus than 2A4CNC, indicating the potential for better shape fidelity. These results are in good agreement with previously reported rheological properties for CNC- and CNF-based bioinks [49,68].

### 3.3. Swelling

Swelling is crucial information for the application of bioink patches because biodegradable patches are usually exposed bodily fluids, such as blood or interstitial fluids, during wound healing [69]. The swelling ratios of 3D printed biodegradable patches as a function of time are shown in Figure 3c. A rapid increase in the swelling is observed after sample immersion, followed by a plateau, as maximum water absorbance is reached. After one hour, the swelling percentage of 4A1CNF is found to be 130%, compared with 102% for 2A4CNC. The 4A1CNF sample reaches its equilibrium swelling capacity of 158% after 4 h. No significant increase is observed after 96 h. 2A4CNC, however, reaches a maximum swelling percentage of 120% after 8 h, with no significant change after the full 96 h. 4A1CNF has a higher alginate concentration and larger pore size compared with 2A4CNC’s, which could explain the increased water absorbance. It could also be attributed to the larger aspect ratio of nanofiber, compared with that of a nanoparticle [70]. CNF-alginate hydrogels have been reported to have higher moisture uptake compared with CNC-alginate hydrogels for both water and PBS [70]. Higher alginate concentrations are also reported to increase swelling percentage [56]. Cellulose nanofibers can be used as an additive material to develop a new patch by customizing its swelling capacity, which may increase the performance of the implantable patch.

### 3.4. Thermal Analysis

Biodegradable patches are typically prepared at room temperature before being implanted, which would cause a temperature difference. Thermal characterization is helpful to estimate the effect of this change on the patch design. Figure 3d shows the results of differential scanning calorimetry (DSC) for both hydrogels. Each hydrogel shows only one endothermic peak (92 °C and 106 °C for 2A4CNC for 4A1CNF, respectively) due to water losses. This endothermic peak is associated with melting temperature. At a body temperature of 37 °C, 4A1CNF has a higher heat flow than 2A4CNC, as seen in Table 1, which may be attributed to the higher thermal conductivity of CNF [57]. Each bioink has one exothermic peak (180–210 °C for 2A4CNC, 205–220 °C for 4A1CNF) due to degradation of the bioinks due to water losses.

Thermogravimetric analysis was used to determine the thermal stability of CNC- and CNF-based scaffolds. Figure 3e depicts three major weight loss steps and thermal degradation. The first step (from 0 °C to 220 °C) is attributed to the water evaporation in the hydrogel structure where 2A4CNC has a gradual 13% loss of mass until 188 °C, while there is 20% weight loss toward 220 °C in 4A1CNF. The slope of the curve at the body temperature of 37 °C, indicated in Table 1, shows 4A1CNF tends towards faster weight loss compared with 2A4CNC. The second step (between 220 and 350 °C) corresponds to the complex degradation and decomposition of glycoside chains in the structure of the hydrogel. In this step, the weight losses are 35% for 2A4CNC and 26% for 4A1CNF up to 350 °C. In the last step, both bioinks have constant weight loss from 350 to 600 °C. Two major degradations occur at 188 °C and at 350 °C to 2A4CNC and at 220 °C and 350 °C to 4A1CNF due to the decomposition of the molecular chain in the component of the bioink formulations. The major degradations in 4A1CNF occur at higher temperatures compared with those of 2A4CNC. This implies that 4A1CNF is more thermally stable than 2A4CNC. This finding agrees with the literature report that CNF-alginate hydrogels are more stable than CNC-alginate hydrogels [70]. Additionally, it has also been reported that the incorporation of either CNF or CNC in pure alginate improves the thermal stability of hydrogel when compared with pure alginate [56,71].

### 3.5. Mechanical Characterization

**Figure 4:**
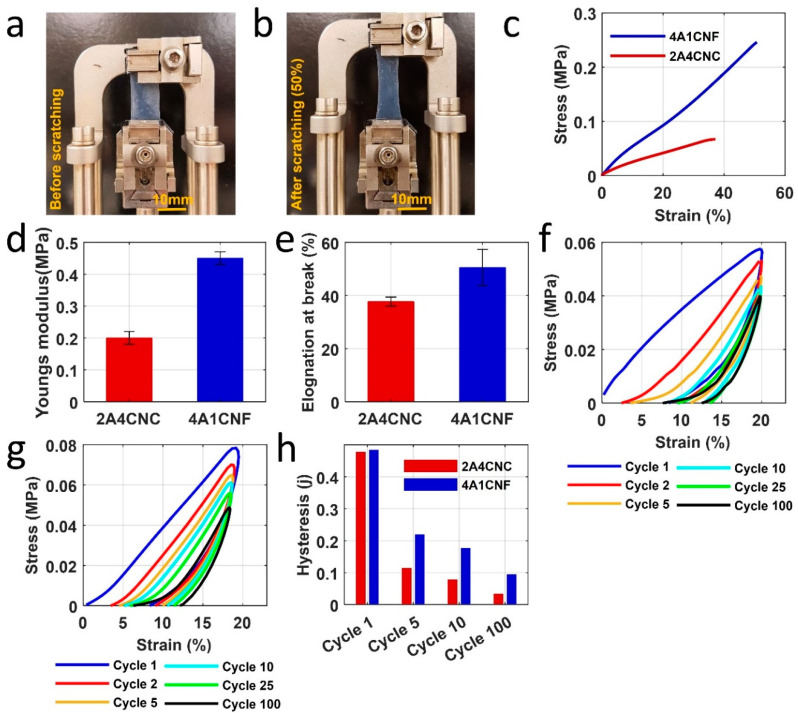
Mechanical characterization of 3D printed patches. (**a**,**b**) Images of 4A1CNF before stretching and 50% elongation under tensile tests. (**c**) Stress-strain curve of two samples under a ramped fore rate of 0.1 N/min. (**d**) Young’s modulus from the slope in the initial linear region of the stress-strain curve for two samples. (**e**) Maximum elongation of sample right before the samples fractured. Cycling loading in DI water to assess the hysteresis, deformation over cycling that mimics the movement in the skin for (**f**) 2A4CNC and (**g**) 4A1CNF. The sample was scratched repeatedly for 100 cycles at strain ramp 10%/min to 20% strain. (**h**) Hysteresis versus the number of cycling from the stress-strain curve of the inside area of loading and unloading.

To help determine how the 3D bioprinted bioinks would perform as flexible and stretchable patches, they are characterized under cycling loading, mimicking the motion of the skin by uniaxial tensile experiment using DMA. Typical stress-strain curves of both samples are shown in Figure 4c. 4A1CNF has higher tensile strength than 2A4CNC. The tensile strength is 0.26 MPa with a failure strain of 50% for 4A1CNF, compared with 0.07 MPa and 38% for 2A4CNC. 4A1CNF is more flexible and more stretchable. Similarly, the average Young’s modulus of 4A1CNF (0.45 ± 0.02 MPa) is much higher than 2A4CNC (0.2 ± 0.02 MPa) (Figure 4d). For human skin experiments, failure strain (30–60%) [72] and Young’s modulus (0.005–140 MPa) are comparable to the results found for the bioinks [73,74]. Elongation at break is shown in Figure 4e. 4A1CNF is able to be stretched 35% larger than 2A4CNC. As the wrist is one of the most flexed parts of the body with a maximum strain of 22%, these results demonstrate that our proposed custom-made patches are adequate for the human body [54].

Both bioinks show relatively linear loading force during the first cycle but nonlinear unloading force over 100 cycles (Figure 4f,g). As restoring strain at zero stress does not return to start after each cycle, proposed patch samples are not 100% elastic and deform during loading. The deformation is not large: reaching a maximum deformation of 10% for 2A4CNC after 20 cycles. Maximum deformation for 4A1CNF is 35% lower than that of 2A4CNC—a mere 6.5% achieved after 10 cycles. No significant additional deformation is observed after the full 100 cycles for either bioink.

Both bioinks have similar hysteresis during the first cycle, around 5 J. There, however, is some reduction after the first cycle and gradually decreases over 100 cycles (Figure 4h). As the samples do not fully restore to their original length, they do not require as much stretching in subsequent cycles.

### 3.6. Evaluation of Degradation

**Figure 5:**
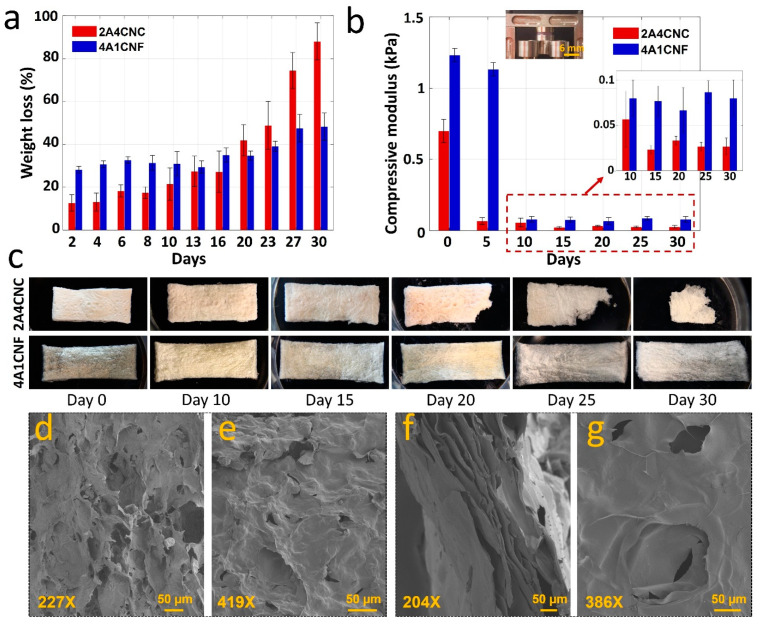
In vitro degradation of printed constructs. (**a**) Weight loss (%) of two samples in cell culture media containing Dulbecco’s modified Eagle’s medium (DMEM) with 10% fetal bovine serum (FBS) over a month. (**b**) The compressive modulus of samples (*l × w × h* = 10 × 10 × 2 mm) was compared with the time of exposure to the cell culture media for 5-day periods up to one month. Inlet: a representative image of a sample undergoing a compression test by a dynamic mechanical analyzer (DMA). Statistical differences were calculated by one-way ANOVA test for multiple comparisons (*p* < 0.0001). (**c**) Pictorial representative images of washed and freeze-dried samples after withdrawal from the media in 5 days period. Error bars represent the standard deviation of 3 independent measurements. Scanning electron microscope (SEM) images of freeze-dried (**d**) 2A4CNC on day 0, (**e**) 2A4CNC after 10-day degradation, (**f**) 4A1CNF on day 0, and (**g**) 4A1CNF after 10-day degradation.

In vitro degradation properties of 2A4CNC and 4A1CNF are summarized in Figure 5. 4A1CNF shows 30% erosion (weight loss) in cell media within the first two days, while 2A4CNC has only 18% degradation over the same time. This is potentially due to the fiber surface of the CNF scaffold, which may be more easily removed compared with 2A4CNC. Following the initial rapid erosion, the weight loss of 4A1CNF slows down and only reaches 35% after 20 days. The weight loss of 2A4CNC increases gradually, reaching 45% after 20 days, reaching 90% at the end of the 30-day degradation trial (Figure 5a). This high degradation can be attributed to the weak chemical bond with the lower alginate concentration in the hydrogel. The 4A1CNF reaches a maximum of 50% after the full 30 days. A similar 50% mass loss over 30 days is reported for the degradation of an alginate-based material [58], with a similar finding reporting that incorporation of CNF in alginate increases resistance against mechanical collapse and degradation. The CNF-alginate hydrogels have better mechanical stability characteristics compared with CNC-alginate hydrogel [70]. Figure 5c shows representative images of washed and freeze-dried samples every 5 days for the duration of the trial. It is worth noting that the 2A4CNC is deformed to degradation, while 4A1CNF maintains most of its shape throughout the degradation.

Mechanical characterization of in vitro degraded samples in the cell culture media is conducted to determine the effect of exposure time on the mechanical properties (Figure 5b). The compressive modulus of the unexposed 2A4CNC and 4A1CNF samples are 0.73 ± 0.08 kPa and 1.2 ± 0.047 kPa, respectively. After degrading for 5 days in media, the compressive modulus of the 2A4CNC decreases to 0.066 ± 0.023 kPa, while there is no large decrease observed in the compressive modulus of the 4A1CNF sample. While the compressive modulus of 4A1CNF decreases sharply to 0.008 ± 0 2kPa over 10 days, it remains 43% higher compared with the compressive modulus of 2A4CNC. Beyond 10 days, there is not any large change in the compressive modulus through the remaining degradation trail. 4A1CNF has around 120 ± 20% larger compressive modulus than 2A4CNC’s through the end of the degradation trials (Figure 5b). The fibrous structure network of 4A1CNF may be more resistant to chemical degradation than the crystalline structure of 2A4CNC. Another potential explanation is that the higher alginate concentration in 4A1CNF forms stronger chemical bonds, providing improved resistance to heat and chemical degradation compared with lower alginate concentrations, as found in the 2A4CNC sample.

Morphological characterization of 2A4CNC and 4A1CNF samples is conducted, observing detailed pore structures and investigating the effects of degradation on the microstructure of samples after 10 days of exposure to media. SEM images show changes in the 2A4CNC’s crystalline structure and fibrous structure of 4A1CNF with degradation (Figure 5d–f). Samples without degradation show porous structures area of 1500 ± 200 µm^2^ for 2A4CNC and 5800 ± 1400 µm^2^ for 4A1CNF. This result is similar to a previously reported study using alginate-CNC hydrogel, where it has been determined that incorporating CNC to pure alginate increases the porosity of hydrogel [52]. A similar finding reported that the addition of CNF to alginate increases the pore size of hydrogel more than the addition of CNC to alginate [70]. Post degradation, the porous structure area reduces to 500 ± 350 µm^2^ for 2A4CNC and 1200 ± 700 µm^2^ for 4A1CNF. After degradation, many pore walls collapse, and pores disappear. Additionally, the SEM images show that CNC and CNF materials homogeneously disperse in the alginate solution.

### 3.7. Cell Viability

**Figure 6:**
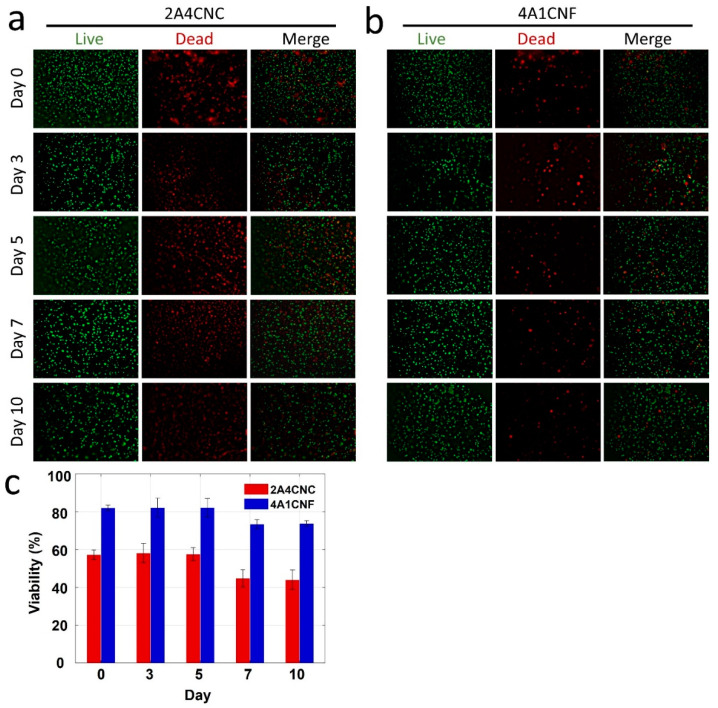
Characterization of cell viability. Fluorescence images of National Institutes of Health (NIH) 3T3 mouse embryonic fibroblast cells in (**a**) 2A4CNC and (**b**) 4A1CNF hydrogels, showing the cell viability after day 0, 3, 5, 7, and 10. The green-stained (Calcein AM, 0.5 µL/mL) cell represents the live-cell shown in the first column. Red-stained (ethidium homodimer 1, 2 µL/mL) cell represents the dead cell shown in the second column. The third column shows the merge of live and dead cells. (**c**) Quantification of the cell viability from live/dead image analysis. Statistical differences were calculated by one-way ANOVA test and *t*-test for multiple and two samples comparison (*p* < 0.0001). Error bars represent the standard deviation of three independent measurements.

NIH/3T3 mouse embryonic fibroblast cells are combined with the hydrogels, and cell viability is observed after ten days using a live/dead assay. Fluorescence images of live/dead cells in 2A4CNC bioink and 4A1CNF bioink are shown in Figure 6a,b, respectively. Cell viability results from live/dead image analysis for two bioinks are summarized in Figure 6c. No significant difference is found in cell viability over the first 5 days of the trials for both bioinks, with average viabilities of 83% and 58% for 4A1CNF and 2A4CNC, respectively (*p* < 0.05 for both). These results are in agreement with previous studies for alginate-CNC [52] and alginate-CNF [75] bioinks. After 5 days, viability decreases across all samples. This decrease is likely due to competition for oxygen and nutrients. Chemical degradation products entering the cell media could also contribute to this decrease, supported by the sharp decrease in compressive modulus on day 5 (Figure 5b). Comparing 4A1CNF to 2A4CNC, the CNF-based bioink shows much higher cell viability across all times, with 43% higher viability on day 5 (*p* < 0.0001). This could be attributed to the higher alginate concentration in the 4A1CNF bioink, which can provide a better environment for the cell. Another reason might be that the 4A1CNF bioink has a higher swelling ratio, which could increase the nutrients available to the cells. 4A1CNF maintains its dimensional and thermal stability, causing high biocompatibility due to the entangled nanofiber network. As a consequence, the present cell viability analysis result shows that the 4A1CNF hydrogel has a great potential for tissue engineering applications.

## 4. Conclusions

We developed two hybrid hydrogels by mixing alginate-cellulose nanocrystal and alginate-cellulose nanofibril and characterized them using our custom-made bioprinter. We optimized parameters for each of the hydrogels for 3D printing, including material concentration, print speed, and nozzle pressure, to achieve the best shape fidelity. CNC has been previously demonstrated for reinforcing alginate-based hydrogels. We demonstrated that CNF as an additive increased the tolerance of the bioink to physical deformation compared with CNC. 4A1CNF had a 10% further elongation before breaking and increased Young’s Modulus to 0.45 MPa compared with 2A4CNC’s 0.2 MPa. Further, the CNF bioink had nearly double the compressive modulus at 1.25 kPa compared with the CNC bioink’s of 0.7 kPa. 2A1CNF was also much more stable under biodegradation conditions, maintaining a weight loss between 30 and 50%, compared with 2A4CNC varying from 14% to 87% over the course of the 30-day experiment. Finally, to validate our proposed materials for tissue engineering, we characterized the cell viability of NIH 3T3 mouse fibroblast cells over time. 4A1CNF demonstrated more than 20% higher cell viability across all times, compared with 2A4CNC. Overall, we demonstrated CNF as a promising additive material for bioink patches because of its better printability, higher mechanical and rheological properties, including viscosity, Young’s modulus, and a compressive modulus. The morphological structure of 4A1CNF had larger porosity, providing its high liquid absorbency and chemical durability against cell media. Besides, it showed excellent chemical and shape fidelity, preserving its shape over the 30-day degradation. Additionally, 4A1CNC exhibited better biocompatibility.

## Figures and Tables

**Table 1:** Thermal characterization results at body temperature of 37 °C. (2A4CNC: 2% Alginate and 4% Cellulose Nanocrystals, 4A1CNF: 4% Alginate and 1% Cellulose Nanofiber).

Scheme	Heat Flow (Watt/gram)	Slope of Weight Loss Curve
2A4CNC	−0.3194	−0.176
4A1CNF	−1.2445	−0.277

## Data Availability

The data presented in this study are available on request from the corresponding author.
